# Prevalence and factors associated with catastrophic health expenditure among slum and non-slum dwellers undergoing emergency surgery in a metropolitan area of South Western Nigeria

**DOI:** 10.1371/journal.pone.0255354

**Published:** 2021-08-31

**Authors:** Taiwo A. Obembe, Jonathan Levin, Sharon Fonn

**Affiliations:** 1 School of Public Health, University of Witwatersrand, Johannesburg, South Africa; 2 Department of Health Policy and Management, Faculty of Public Health, College of Medicine, University of Ibadan, Ibadan, Nigeria; Institute for Human Development, INDIA

## Abstract

**Background:**

Out of Pocket (OOP) payment continues to persist as the major mode of payment for healthcare in Nigeria despite the introduction of the National Health Insurance Scheme (NHIS). Although the burden of health expenditure has been examined in some populations, the impact of OOP among slum dwellers in Nigeria when undergoing emergencies, is under-researched. This study sought to examine the prevalence, factors and predictors of catastrophic health expenditure amongst selected slum and non-slum communities undergoing emergency surgery in Southwestern Nigeria.

**Methods:**

The study utilised a descriptive cross-sectional survey design to recruit 450 households through a multistage sampling technique. Data were collected using pre-tested semi-structured questionnaires in 2017. Factors considered for analysis relating to the payer were age, sex, relationship of payer to patient, educational status, marital status, ethnicity, occupation, income and health insurance coverage. Variables factored into analysis for the patient were indication for surgery, grade of hospital, and type of hospital. Households were classified as incurring catastrophic health expenditure (CHE), if their OOP expenditure exceeded 5% of payers’ household budget. Analysis of the data took into account the multistage sampling design.

**Results:**

Overall, 65.6% (95% CI: 55.6–74.5) of the total population that were admitted for emergency surgery, experienced catastrophic expenditure. The prevalence of catastrophic expenditure at 5% threshold, among the population scheduled for emergency surgeries, was significantly higher for slum dwellers (74.1%) than for non-slum dwellers (47.7%) (F = 8.59; p = 0.019). Multiple logistic regression models revealed the significant independent factors of catastrophic expenditure at the 5% CHE threshold to include setting of the payer (whether slum or non-slum dweller) (p = 0.019), and health insurance coverage of the payer (p = 0.012). Other variables were nonetheless significant in the bivariate analysis were age of the payer (p = 0.017), income (p<0.001) and marital status of the payer (p = 0.022).

**Conclusion:**

Although catastrophic health expenditure was higher among the slum dwellers, substantial proportions of respondents incurred catastrophic health expenditure irrespective of whether they were slum or non-slum dwellers. Concerted efforts are required to implement protective measures against catastrophic health expenditure in Nigeria that also cater to slum dwellers.

## Introduction

Catastrophic health expenditure (CHE) has been defined as spending on health care that exceeds a certain proportion of the patient’s income [1, 2]. It has been observed to be real and sizeable in both rich and poor countries [3]. This, in turn, leads to a continuation in the chain of urban slum-dweller poverty and ill health, a situation almost always linked to worse health outcomes [4]. During illness episodes, families may opt for less costly traditional, sub-optimal care, or altogether forgo healthcare services they need [5, 6]. Having to meet health care costs can pose substantial threats to the provision of basic household necessities such as food, clothing and shelter [7]. In extreme conditions, the need to pay for medical care can make education unaffordable [8]. Having to make these kinds of choices has led to the coining of the term catastrophic health care expenditure. Catastrophic health expenditure has been evaluated in several studies and also at different thresholds, at 5%, 10%, 25% and 40% of household budget [9, 10]. Others advocate the use of 10% of all household expenditure [11, 12] or 40% of non-food consumption expenditure [13, 14]. The range of CHE differs greatly among many low- and middle-income countries (LMIC). Proportion of CHE ranges from as low as 9.1% in India [15] to as high as 25.0% in Nigeria [10]. The risk and occurrence of exorbitant (otherwise catastrophic) expenditures when a health need arises defies the concept of universal health coverage (UHC) [16, 17].

Universal health coverage (UHC) remains an important component for positive health outcomes and a right to access quality health care is a basic human right throughout the world today [16]. Nigeria appears to support the view that health care should be accessible and affordable for all. Nonetheless, disparities in access to health care continue to persist despite promising national health policies. The current health insurance scheme in Nigeria, the National Health Insurance Scheme (NHIS), was implemented in 2005 to offer financial protection for its citizens [18, 19]. Despite this, the NHIS to date only covers the formal [through the Formal Sector Social Health Insurance (FSSHIP)] and organized private sectors which comprise 4.0% of the Nigerian population, and excludes the informal sector [20]. One group, "*urban slum dwellers*", constitute an important part of the informal sector and may be more vulnerable to illnesses.

Worldwide, it is estimated that between 2000 and 2010, the number of slum dwellers has increased by over 50 million [21]. Slums are rapidly forming in many cities today due to urbanization and population growth that attracts migrants in search of economic opportunities. Currently, approximations show that one out of three urban dwellers (one out of every six people worldwide) live in a slum [22]. Residents of slums are subjected to a reduced access of basic sanitation and poor urban or regional planning facilities, which may lead to unprecedented health problems compared to non-slum dwellers. This increased risk of ill-health also places slum dwellers at greater risk of catastrophic payments when they fall ill, as they are left to pay for hospital bills via out-of-pocket (OOP) payments.

In Nigeria, OOP expenditure as a percentage of private expenditure on health was reported to be higher than 90% in 2002 and it increased to 95.7% in 2012 [23]. The Nigerian health care system is typically structured in three tiers–tertiary, secondary and primary (Ward Health System operating at the local government level). At the secondary level of care, there is a wider array of private and public hospitals. Public hospitals are not-for-profit, usually multidisciplinary, bigger in terms of size and built to serve larger populations. They have a wider array of human resources (core medical team and allied health workers such as radiographers, pharmacists together) and offer both general and specialist services. Private hospitals, on the other hand are usually set up as specialist hospitals in which the services rendered have expertise in a certain field/specialty of medicine [24, 25]. They are usually for-profit, smaller (thus have fewer allied staff) and built to attend to fewer patients.

General factors that have been identified to influence a preferred choice of facility to access healthcare range from availability of essential drugs, user fees, proximity of facility, cleanliness of the environment, to the reputation of the facilities (quality of services) [26]. From literature, we observe that perceptions of shorter waiting times, flexible access and greater confidentiality are factors that might draw consumers to private facilities compared to public health care facilities [25]. Hospitals that are for-profit (private) or not-for-profit (public) have different approaches to payments and flexibility of payment. This influences preferences, satisfaction, access to care and their ability to pay [19, 27].

**Fig 1:**
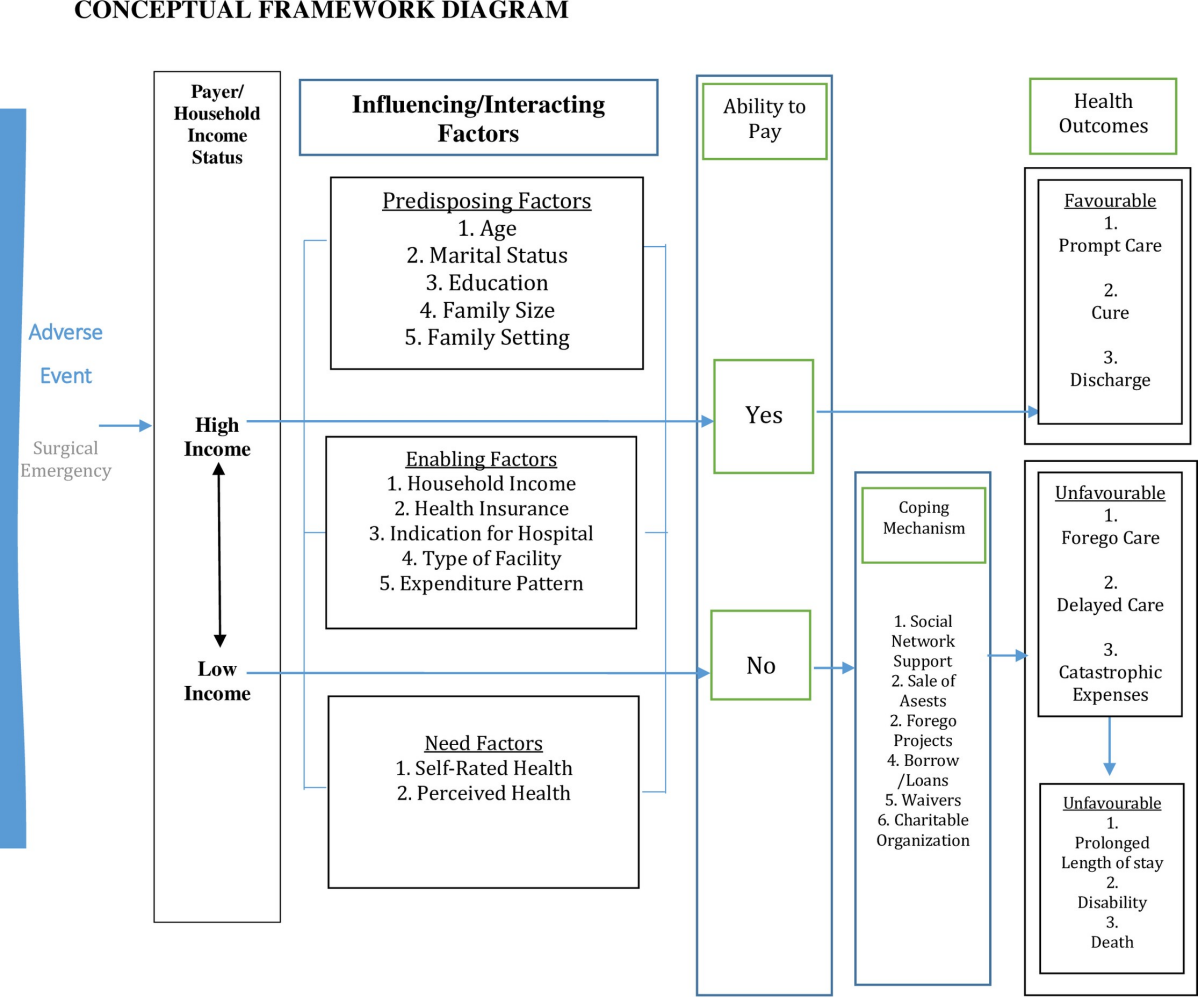
Conceptual framework for patterns of expenditure, coping mechanism among households admitted for emergency surgeries in Ibadan, Nigeria. Adapted from Wilkes et al. (1997) [30] pp3; Tseng & Khan 2015 [31] pp3 and Wild & Hanes (1976) [32].

A particularly acute example is that of an event that requires emergency surgical care [28]. Such events are usually sudden and unplanned [29]. Certain factors interact with the individual’s circumstances that either favour or hinder the ability of a household to afford the hospital costs. Sometimes, one or more of these factors can influence the household’s ability to pay [30–32]. They include socioeconomic status (such as the income of the household) and influencing/interacting factors (that maybe predisposing, enabling or need factors) (Fig 1).

An individual with a decent income will probably be able to afford the payment, thus increasing the likelihood of enjoying favourable outcomes. Conversely, a household with poor income is more likely to experience some difficulty with payment or affordability of health care costs. Detention and incarceration of patients within the hospital premises until payments have been paid is a common practice in many sub-Saharan African hospitals [33–35]. The inability to pay on time invariably predisposes the individual to unfavourable or unpleasant outcomes.

Many studies have explored the risks of catastrophic expenditures and impoverishment in emergencies among several population [20, 36–39] in different populations. However, a paucity of data exists with respect to the impact of OOP health care expenditure on slum dwellers in emergency conditions. A research conducted in Nigeria explored the influence of OOP healthcare payment on Caesarian Section (CS) [40], however, neither the residence (whether slum/non-slum) nor catastrophic nature of the health care expenses were explored. Furthermore, there is a dearth of information on a comparative assessment of catastrophic health spending among slum and non-slum dwellers in the sub-Saharan Africa. Given an increased exposure to catastrophic payments when the medical need is financed on an out-of-pocket basis and the fact that urban slum dwellers constitute a population that is exposed to a higher risk of illness, it thus becomes imperative to study this micro-unit’s experience of CHE. This study thus sought to answer the research question, “Is there a significant difference in catastrophic health spending between slum and non-slum dwellers when faced with a surgical emergency?”

Using emergency surgery as a lens, because of its peculiar characteristics of being unplanned, sudden and often unavoidable, this study examined the relationship between emergency surgery and catastrophic health expenditure among the urban slum and non-slum dwellers in a metropolis of Southwestern Nigeria.

## Methods

The study utilized a cross-sectional design involving 450 respondents from both slum and non-slum areas. Data were collected using validated interviewer-administered questionnaires. This study took place in Ibadan, the state capital of Oyo State. It is situated in the southwest geo-political zone of Nigeria with a landmass of 28,246.264 km^2^ and a population of approximately 6,182,000 [41]. Oyo State consists of 33 local government areas (LGAs) that function as administrative units out of which 5 of the 33 local government areas make up the State capital (Ibadan). At the time of data collection, there were a total of 79 primary health care centres, 197 secondary health facilities and one tertiary facility (University College Hospital) [35] across the 5 local government areas.

A multistage sampling technique was employed for the recruitment of study respondents. The urban slums are located within these 5 local government areas (Ibadan North, Ibadan North-East, Ibadan North-West, Ibadan South-West, and Ibadan South East) [42]. Within these LGAs the main unit of selection was the hospitals that served the population of the chosen LGA. A list of all health care facilities located within the metropolis was obtained. All primary health care were removed based on the fact that they could not offer surgical services. The remaining health facilities on the list were stratified as secondary/tertiary (grade of hospital), public/private (type of hospital), thus an equal representation of the different types of facilities were selected.

**Fig 2:**
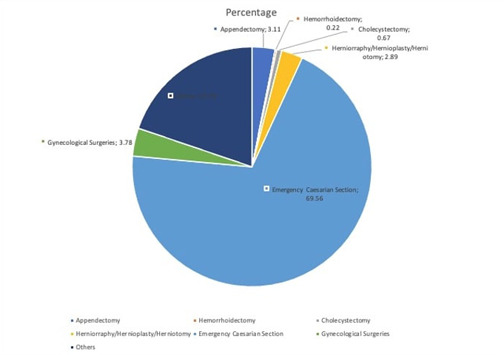
Indications for surgery. ⸸Others: Lumpectomy, Orchidectomy, Lipomas, Splenectomy, Hydrocelectomy, Fistulectomy, Cystectomy.

In the tertiary stratum, the only tertiary facility within the 5 LGAs was purposively selected [43]. After an arrangement of all secondary facilities were listed out, a sample of six public and nine private secondary facilities were randomly sampled across the five LGAs. In each of the selected secondary health facilities, four departments that could offer emergency surgical services were identified (Departments of Surgery, Casualty, Obstetrics and Gynaecology) per facility. The register of patients from each of these departments was used to estimate the sample frame per facility one month prior to the start of our data collection. (The patients were however, merely a proxy for identifying the payers that constituted the study population). Using proportional allocation, the maximum number of patients to be recruited per facility was calculated. This number was divided into four to ensure that an equal number of patients were recruited equally from the four departments above. Thereafter, recruitment was carried out systematically after the patients were screened for eligibility (such as had met the inclusion criteria for the study). In order to ensure randomization, every other patient (representing a household) that met the inclusion criteria were approached to participate in the study. If a patient declined to participate, the next eligible patient was approached and this continued systematically until the estimated sample per department and facility was cumulatively attained. Patients that were admitted with an anticipated hospital admission duration exceeding one month (such as orthopedic and neurological surgeries) were excluded from the study. The surgeries that were qualified for enrollment into the study were analysed under two main categories—emergency caesarian section and others (appendectomies, hemorrhoidectomies, cholecystectomies, herniorrhaphies, hernioplasties, herniotomies, and minor gynecological surgeries as itemized in Fig 2).

In order to ensure that payer’s income was captured without any ambiguity, payers had to confirm that they were solely and exclusively responsible for payment of hospital bills for the patient and that hospital costs were not shared with any other family member.

It was estimated that a sample of 300 respondents (150 slum dwellers and 150 non-slum dwellers) would give 80% power to detect an absolute difference of 15% in the proportion of respondents that incur CHE between slum dwellers and non-slum dwellers at the 5% significance level. This was calculated assuming that approximately 23% of the non-slum dwellers would experience CHE as found in a Kenyan study [8]. (Finite population correction was applied to the sample size estimation). Recruitment for both the slum and non-slum dwellers was based on having a family member scheduled for emergency surgery. In this study, we defined emergency surgery as any ‘surgical procedure that cannot be delayed, for which there is no alternative therapy, and for which a delay could result in death or permanent impairment of health’ [44]. We also defined slum as a compact area with 300 residents or which had 60–70% of the households having poorly congested rooms with inadequate infrastructure, lack of proper sanitation and drinking water facilities [45]. Study respondents were those responsible for paying the hospital bills (referred to as payers in this study) of a household member (patient) admitted for emergency surgery. The respondents (payers) had to be above 18years and resident for at least 12 months before the study in either the slum or non-slum areas respectively. In addition to being able to legally consent to participate in the study, payers also had to provide consent for a 1-month follow-up at the recruitment phase. To allow for a loss of precision due to clustering effects, a total of 500 respondents were approached.

Fifty respondents declined, yielding a response rate (RR) of 90%. Those that declined included households without a definitive payer. Others that declined to participate included those that were psychologically assessed as unfit to provide consent or credible responses. Thus, a total of 450 respondents were eventually recruited for the study. Families with a household member (patient) admitted for emergency surgery were approached and the recruitment was carried out sequentially as patients presented for emergency surgery until the sample size requirement was met for each group. Non-financial incentives were provided for all respondents that were successfully recruited to participate in the study. The incentives included basic items that were considered to be useful while on admissions such as face towels and detergents. Also, a vehicle was made available to facilitate hospital-related errands for families that requested the service.

An interviewer-administered instrument adapted from the World Health Survey household questionnaire [46], consisting of a baseline survey and cost diary, was utilised to estimate sociodemographic and baseline data as well as health costs during admission. Three types of questions were utilised; binary questions (such as yes/no); multiple-choice questions (in which the options were mutually exclusive and covered all possible answers); specific open-ended questions (for example how old are you?) [47]. The Gunning Fog ease score of 9.4 and the Coleman-Liau index of 9 (indicating that it was fairly easy to read) were used to ensure proper comprehension of the instrument [48, 49]. A pre-test of the questionnaire was carried out among 50 respondents from similar secondary and tertiary health facilities located in slums outside the study area before the actual data collection. All income and expenditure data were collected in Naira (N) between May 2017 and August 2017.

### Main variable construction

Our explanatory variables included setting (whether slum or non-slum dweller), gender (of payers), age (of payers), indication for surgery (for patients), educational status (of the payer), marital status (of the payer), ethnicity (of the payer), religion (of the payer), occupation (of the payer), income (of the payer), type of hospital (private or public), grade of hospital (secondary or tertiary) and health insurance coverage (of the payer) while the outcome variable was the “presence or absence of CHE”. The “*proportionality of income”* approach was used to examine for catastrophic expenditure [8]. A respondent was deemed to have incurred CHE if OOP expenditure on health as a fraction of payer’s annual income exceeded the specified threshold which was set at 5% for the main analysis [50]. The threshold of 5% was chosen for two main reasons. Firstly, this threshold has been used for some studies exploring catastrophic expenditure. Secondly, it was also chosen to examine the prevalence of CHE at the lowest possible threshold possible to allow for comparison of findings across a wide range of thresholds that have been documented in literature [51, 52]. A sensitivity analysis was thereafter carried out in which predictors of CHE were assessed at three other cut-offs (10%, 25% and 40%).

To calculate the health costs, information was collected for hospital registration, hospital consultation, investigations, drugs, in-patient hospital stays, transportation costs directly related to admission and other costs related to admission. All costs related to the index admission whether they were on the hospital premises were all captured and added together. For instance, drugs purchased from pharmacies outside the hospital premises were considered and added together as total costs spent on drugs. These were added to make up the total health expenditure (*Ti*) incurred for the admission. To estimate the annual household income (x_i_), the monthly income over the past 3 months was requested from all income earners within the household (cumulatively summed up as the income of the payer). An average was estimated for those respondents without a steady or formal source of income. Daily and weekly paid respondents were converted to monthly income using an appropriate multiplier (26 days or 4 weeks respectively, to exclude Sundays). The annual income was calculated by multiplying the estimated monthly income by 12. Catastrophic health expenditure was constructed as *‘not catastrophic = 0*”; *“catastrophic = 1”* for the bivariate analysis and the logistic regression.

Associations between the outcome variable (categorized presence or absence of CHE) and the explanatory variables were investigated at the 5% level of significance. Due to the multi-stage sampling design, the analysis accounted for both the clustering of respondents within a hospital and the differential probabilities of selection for different respondents through the use of probability weights. In the bivariate analysis, clustering of respondents within a hospital was accounted for using the Rao Scott adjustment to the Pearson chi-square test to investigate the associations between the outcome and the explanatory variables. Frequency tables were generated for relevant variables while descriptive statistics were used to summarize categorical variables. Logistic regression models that accounted for the survey design were fitted to identify the independent predictors of CHE. All variables in the bivariate analysis were fitted into the logistic regression model and presented as unadjusted and adjusted odd ratios. For this study, we report the model where CHE was based on payer’s income. At the time when data was collected, the sum of N360 was equivalent to $1.

Approvals to conduct the study were obtained from the Human Ethics Review Committee of University of Witwatersrand Johannesburg (M170284), University of Ibadan/University College Hospital (UI/UCH) Ethical committee (UI/EC/17/0006) and Oyo State Ministry of Health (AD13/479/123). Written informed consent was obtained from all patients and payers (above 18 years of age) before questionnaire administration while assent was also obtained from patients below 18 years. Respondents’ anonymity was protected by ensuring that no individual identifiers existed in the instruments or the electronic data set.

## Results

Table 1 summarizes the characteristics of the study participants that constituted the slum and non-slum populations. Among slum dwellers, over two-thirds (78.2%) of the payers are male. The same was observed among the non-slum dwellers where 73.8% of the payers were male compared to 26.2% female. For both slum and non-slum dwellers, most of our payers were aged less than 40 years (65.6% among slum dwellers and 63.9% among non-slum dwellers); currently married (85.1% among slum dwellers and 93.4% among non-slum dwellers); Yoruba (65.5% among slum dwellers and 78.9% among non-slum dwellers) and Christians (64.4% among slum dwellers and 68.1% among the non-slum dwellers). Significantly more (48.2%) of the non-slum dwellers, attained a level of education that was higher than secondary level compared to the slum dwellers, where only 16.0% had education above the secondary level (p<0.001). Emergency CS accounted for the commonest indication why patients were hospitalised for both slum (82.0%) and non-slum dwellers (83.5%) (Table 1). A breakdown of the indications as presented in Fig 2. More payers from the slums were employed in the informal sector (50.3%) while payers from non-slum dwellers were employed in the formal sector (53.0%) and this was observed to be significant (p = 0.0025). Earning capacity between slum and non-slum dwellers was also found to be very significant (p<0.001). Income greater than $1,389 (N500,000) per annum was found to be common among the non-slum dwellers (71.0%) compared to slum dwellers whose income was less than $1,389 (N500,000) (71.1%). The proportion of respondents with health insurance coverage was low among the slum dwellers (11.3%) and non-slum dwellers (9.3%) (Table 1).

**Table 1:** Selected characteristics of respondents and patterns of healthcare access.

	Variable N = 450	Slum n (%)	Non-Slum n (%)	⸸RS F	p-value
**1**	**Sex of Payer**				
	Male	171 (78.2)	135 (73.8)	0.4208	0.5347
	Female	68 (21.8)	76 (26.2)		
**2.**	**Age of payer**				
	<39 years	144 (65.6)	109 (63.9)	0.0246	0.8792
	≥40 years	95 (34.4)	102 (36.1)		
**3.**	**Indication for surgery(patient)**				
	Emergency CS‡	170 (82.0)	143 (83.5)	0.0638	0.8069
	Abdominal surgeries & Others	69 (18.0)	68 (16.5)		
**4.**	**Educational status of the payer**				
	Above secondary	67 (16.0)	132(48.2)	51.6718	0.0001*
	Below Secondary	172 (84.0)	79 (51.8)		
**5.**	**Marital Status of the payer**				
	Not currently married	38 (14.9)	21 (6.6)	18.0639	0.0028*
	Currently married	201 (85.1)	190 (93.4)		
**6.**	**Ethnicity of the payer**				
	Yoruba	155 (65.5)	161 (78.9)	2.9678	0.1232
	Others#	84 (34.5)	50 (21.1)		
**7.**	**Religion of the payer**				
	Christianity	156 (64.4)	141 (68.1)	1.3155	0.2845
	Islam	83 (35.6)	70 (31.9)		
**8.**	**Occupation of the payer**				
	Formal	76 (31.3)	120 (53.0)	12.8024	0.0025*
	Informal	120 (50.3)	86 (46.3)		
	Unemployed	43 (18.4)	5 (0.7)		
**9.**	**Income of the payer**				
	≤ $1,389 (N500,000)	206 (71.1)	50 (29.0)	51.8795	0.0001*
	>$1,389 (N500,000)	33 (28.9)	161 (71.0)		
**10.**	**Type of hospital**				
	Private	94 (89.2)	48 (77.1)	0.4248	0.5328
	Public	145 (10.8)	163 (22.9)		
**11.**	**Grade of Hospital**				
	Secondary	162 (96.2)	93 (87.8)	1.4024	0.2703
	Tertiary	77 (3.8)	118 (12.2)		
**12.**	**Health Insurance**				
	Yes	27 (11.3)	25 (9.3)	0.5854	0.4662
	No	212 (88.7)	186 (90.7)		

# = Igbo, Hausa

‡ CS = Caesarian Section.

⸸RS F = Rao Scott Statistic

### Bivariate results

The bivariate analysis (Table 2) presents results that shows relationships between the explanatory variables and the outcome variables [(occurrence of catastrophic expenditure CHE+) or no occurrence CHE-)]. Overall, 65.6% (95% CI: 55.6–74.5) of participants experienced CHE among both slum and non-slum populations combined. The prevalence of catastrophic expenditure observed at the 5% threshold was significantly higher among slum dwellers (74.1%) than for non-slum dwellers (47.7%) (Rao-Scott F = 8.59; p = 0.019). Variables that were significantly related to CHE at the 5% threshold were setting of residence of the participant (whether slum or non-slum) (Rao-Scott F = 8.59, p = 0.019), age of payer (F = 8.95, p = 0.017), marital status (F = 8.048, p = 0.022), income (F = 105.39, p<0.001) and health insurance coverage (F = 10.41, p = 0.012). Among those with health insurance coverage, only 21.1% experienced CHE whereas 70.9% among those without any form of coverage experienced catastrophic expenditure (Table 2).

**Table 2:** Cross tabulation of selected characteristics and proportion of CHE at 5%, 10%, 25% and 40%.

S/No	Variable (N = 450)	CHE+ 5%	⸸RS F	p-value	CHE+ 10%	⸸RS F	p-value	CHE+ 25%	⸸RS F	p-value	CHE+ 40%	⸸RS F	p-value
1	Setting												
	Slum	208(74.11)	8.593	0.019*	201(71.73)	65.688	<0.001*	182(66.47)	109.01	<0.001*	159 (57.89)	44.009	<0.001*
	Non-Slum	103(47.67)			79 (36.32)			42(21.56)			27 (15.37)		
2	Sex of payer												
	Male	208(65.21)	0.042	0.842	194(60.08)	0.017	0.898	158(51.29)	0.188	0.676	133 (44.57)	0.136	0.721
	Female	103(67.05)			86(61.34)			66(54.62)			53 (43.21)		
3	Age of payer												
	<39 years	184(72.13)	8.95	0.017*	165(65.82)	3.24	0.109	142(59.31)	7.142	0.029*	119 (49.54)	4.706	0.062
	≥40 years	127(53.56)			115(50.25)			82 (38.6)			67 (34.43)		
4	Indication for surgery(patient)												
	Emergency CS‡	209 (63.22)	1.697	0.229	191(58.33)	2.249	0.172	154(50.78)	0.765	0.407	125 (41.92)	1.429	0.266
	Abdominal surgeries & Others	102 (76.98)			89(69.99)			70(58.10)			61 (55.26)		
5	Educational status of the payer												
	Above secondary	121(59.30)	0.393	0.548	105(52.01)	0.9144	0.367	75(42.33)	1.309	0.286	61 (32.28)	3.734	0.089
	Below Secondary	190 (67.9)			175(63.36)			149(55.55)			125 (48.54)		
6	Marital Status of the payer												
	Not currently married	40 (45.97)	8.048	0.022*	36(44.89)	3.016	0.121	30(37.04)	10.263	0.013*	24 (34.33)	7.687	0.024*
	Currently married	271(68.38)			244(62.53)			194(54.16)			162 (45.64)		
7	Ethnicity of the payer												
	Yoruba	218(69.29)	2.984	0.122	193(62.08)	0.861	0.381	150(52.84)	0.471	0.512	125 (47.29)	3.706	0.090
	Others#	93(57.19)			87(56.42)			74(50.28)			61 (37.25)		
8	Religion of the payer												
	Christianity	198(63.22)	2.286	0.169	181(57.07)	1.754	0.222	139(50.3)	0.337	0.578	116 (42.68)	0.526	0.489
	Islam	113(70.23)			99(66.67)			85(55.43)			70 (47.26)		
9	Occupation of the payer												
	Formal	126(60.87)	2.433	0.145	117(58.18)	5.109	0.030*	86(47.96)	4.534	0.036*	71 (37.49)	11.628	0.007*
	Informal	142(62.18)			122(53.68)			98(46.12)			83 (42.01)		
	Unemployed	43(93.24)			41(92.72)			40(87.27)			32 (73.24)		
10	Income of the payer												
	≤ $1,389 (N500,000)	243 (97.99)	105.39	<0.001*	234 (96.2)	136.55	<0.001*	209 (88.33)	673.60	<0.001*	178(76.43)	417.99	<0.001*
	>$1,389 (N500,000)	68 (21.76)			46 (11.78)			15 (2.89)			8 (0.63)		
11	Type of hospital												
	Private	91(64.72)	0.198	0.668	82(59.72)	0.074	0.793	71(52.32)	0.009	0.927	61 (44.96)	0.080	0.785
	Public	220(70.98)			198(64.17)			153(50.57)			125 (40.14)		
12	Grade of Hospital												
	Secondary	172 (65.24)	0.919	0.366	158(60.22)	0.082	0.783	134(52.48)	0.262	0.623	107 (44.52)	0.087	0.776
	Tertiary	139 (71.28)			122(62.56)			90(46.15)			79 (40.51)		
13	Health Insurance												
	Yes	23 (21.1)	10.407	0.012*	16 (12.8)	18.27	0.003*	12 (11.5)	10.194	0.013*	9 (9.64)	8.483	0.020*
	No	288 (70.9)			264 (66.1)			212 (56.9)			177 (48.4)		

**Proportions are weighted row percentages

⸸RS F: Rao Scott Statistic

# = Igbo, Hausa, CHE-: Not Catastrophic; CHE+: Catastrophic.

### Predictors of CHE (unadjusted and adjusted results of logistic regression model)

Tables 3 and 4 presents the unadjusted and adjusted results of fitting multiple logistic regression models to the outcome of catastrophic expenditure (accounting for the two-stage survey design). Predictors found to be significant at all four thresholds in the unadjusted analysis (Table 3) were setting of residence (SD/NSD), occupation of the payer, income of the payer and health insurance coverage of the payer. Age of payer was significant at 5% and 25% thresholds only while marital status was significant at 5%, 25% and 40%. However, in the adjusted analysis (Table 4), only income was a significant predictor across the four thresholds that was examined. Other predictor variables found to be significant at one or more thresholds were setting of residence (at 25% and 40% thresholds only), sex of payer (at 25% threshold only), age of payer (at 5% and 25% thresholds only), occupation of payer (at 10% threshold only), grade of hospital (at 10% threshold only), health insurance (at 10% and 25% thresholds only).

**Table 3:** Unadjusted Odds Ratios (UOR) for respondents at all thresholds.

		5% Threshold			10% Threshold			25% Threshold			40% Threshold		
S/No	Variable (N = 450)	UOR	P-value	CI	UOR	P-value	CI	UOR	P-value	CI	UOR	P-value	CI
1	Setting												
	Slum	1			1			1			1		
	Non-Slum	0.32	0.021*	0.13–0.79	0.23	<0.001*	0.15–0.35	1.39	<0.001*	0.087–0.221	0.13	<0.001*	0.06–0.28
2	Sex of payer												
	Male	0.92	0.84	0.37–2.32	0.95	0.89	0.38–2.39	0.88	0.676	0.43–1.78	1.06	0.721	0.75–1.49
	Female	1			1			1			1		
3	Age of payer												
	<39 years	2.24	0.018*	1.20–4.21	1.91	0.11	0.83–4.38	2.31	0.029*	1.12–4.82	1.87	0.063	0.96–3.65
	≥40 years	1			1			1			1		
4	Indication for surgery(patient)												
	Emergency CS‡	1			1			1			1		
	Abdominal surgeries & Others	1.95	0.234	0.59–6.41	1.67	0.18	0.76–3.67	1.34	0.41	0.62–2.94	1.71	0.27	0.60–4.86
5	Educational status of the payer												
	Above secondary	1			1			1			1		
	Below Secondary	1.45	0.55	0.37–5.75	1.59	0.37	0.52–4.95	1.70	0.29	0.58–5.00	1.98	0.092	0.87–4.50
6	Marital Status of the payer												
	Not currently married	1			1			1			1		
	Currently married	2.54	0.024*	1.17–5.52	2.05	0.13	0.78–5.39	2.01	0.013*	1.21–3.34	1.61	0.025*	1.08–2.39
7	Ethnicity of the payer												
	Yoruba	1.69	0.124	0.84–3.41	1.27	0.38	0.71–2.27	1.11	0.512	0.79–1.56	1.51	0.091	0.92–2.48
	Others#	1			1			1			1		
8	Religion of the payer												
	Christianity	1			1			1			1		
	Islam	1.37	0.17	0.85–2.23	1.51	0.22	0.74–3.07	1.23	0.58	0.54–2.79	1.2	0.489	0.67–2.17
9	Occupation of the payer												
	Formal	0.11	0.013*	0.02–0.55	0.11	0.009*	0.03–0.49	0.13	0.012*	0.03–0.56	0.22	0.005*	0.09–0.55
	Informal	0.12	0.064	0.01–1.17	0.09	0.018*	0.014–0.58	0.125	0.016*	0.02–0.60	0.27	<0.001*	0.17–0.42
	Unemployed	1			1			1			1		
10	Type of hospital												
	Private	1			1			1			1		
	Public	1.33	0.67	0.30–5.94	1.21	0.79	0.24–6.01	0.93	0.92	0.17–5.14	0.82	0.785	0.16–4.14
11	Grade of Hospital												
	Secondary	1			1			1			1		
	Tertiary	1.32	0.37	0.67–2.59	1.10	0.78	0.49–2.45	0.78	0.62	0.25–2.44	0.85	0.776	0.24–3.07
12	Health Insurance												
	Yes	0.11	0.023*	0.02–0.68	0.075	0.008*	0.14–0.42	0.09	0.028*	0.13–0.72	0.11	0.038*	0.02–0.86
	No	1			1			1			1		
13	Income of the payer⊥												
	≤ $1,389 (N500,000)	174.96	<0.001*	28.97–1056.94	189.75	<0.001*	46.29–777.86	254.39	<0.001*	119.67–540.86	514.4	<0.001*	96.23–2749.85
	>$1,389 (N500,000)	1			1			1			1		

UOR: Unadjusted Odds Ratios; CI = 95%Confidence Intervals

**Fig 3:**
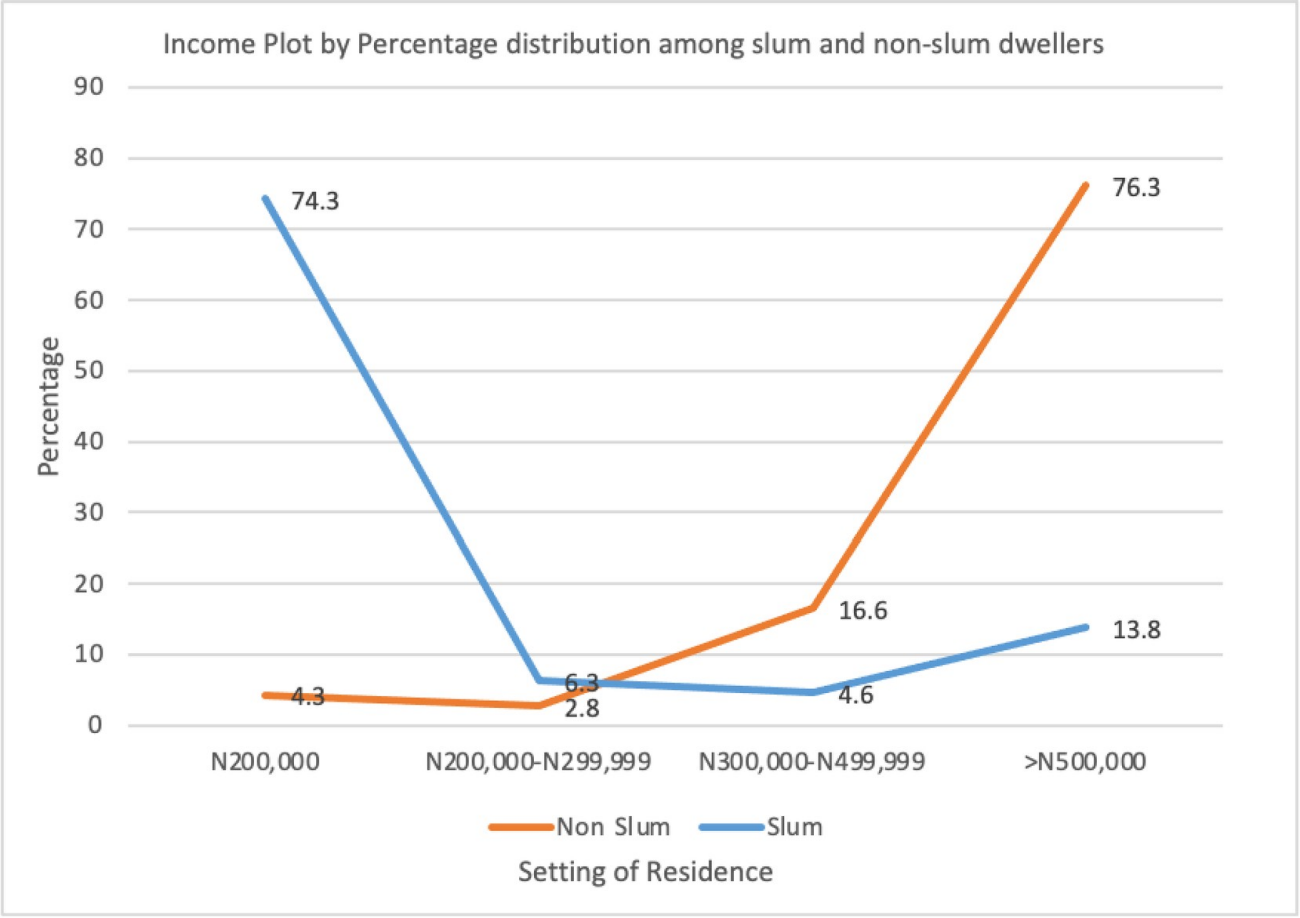
Income plot by percentage distribution among slum and non-slum dwellers.

*Significant Associations, ⊥-Income UOR have wide CI due to the skewed distribution (Fig 3).

‡CS = Caesarian Section; # = Igbo, Hausa

**Table 4:** Adjusted Odds Ratios (AOR) for respondents at all thresholds.

		5% Threshold			10% Threshold			25% Threshold			40% Threshold		
S/No	Variable (N = 450)	AOR	P-value	CI	AOR	P-value	CI	AOR	P-value	CI	AOR	P-value	CI
1	Setting												
	Slum (ref)	1			1			1			1		
	Non-Slum	2.82	0.42	0.17–47.96	1.03	0.97	0.18–5.88	0.16	0.001*	0.07–0.39	0.15	0.002*	0.06–0.40
2	Sex of payer												
	Male	0.87	0.91	0.07–11.49	0.65	0.69	0.06–7.14	0.22	0.009*	0.08–0.61	1.12	0.859	0.28–4.55
	Female (ref)	1			1			1			1		
3	Age of payer												
	<39 years	3.51	0.005*	1.63–7.54	1.79	0.36	0.45–7.20	2.98	0.017*	1.29–6.86	1.39	0.381	0.61–3.16
	≥40 years (ref)	1			1			1			1		
4	Indication for surgery(patient)												
	Emergency CS‡ (ref)	1			1			1			1		
	Abdominal surgeries & Others	1.55	0.649	0.18–13.12	1.24	0.83	0.13–11.82	0.52	0.40	0.96–2.84	1.26	0.718	0.30–5.22
5	Educational status of the payer												
	Above secondary (ref)	1			1			1			1		
	Below Secondary	2.78	0.197	0.52–14.92	2.00	0.43	0.29–13.39	1.50	0.60	0.27–8.43	2.13	0.356	0.36–12.74
6	Marital Status of the payer												
	Not currently married (ref)	1			1			1			1		
	Currently married	1.63	0.46	0.38–7.06	0.63	0.74	0.03–14.35	1.60	0.43	0.43–5.95	0.38	0.114	0.11–1.34
7	Ethnicity of the payer												
	Yoruba	2.02	0.23	0.58–6.99	0.83	0.78	0.19–3.67	0.82	0.70	0.25–2.67	1.98	0.239	0.57–6.91
	Others# (ref)	1			1			1			1		
8	Religion of the payer												
	Christianity (ref)	1			1			1			1		
	Islam	1.82	0.49	0.27–12.13	2.59	0.19	0.54–12.42	1.53	0.66	0.18–13.05	1.68	0.318	0.55–5.19
9	Occupation of the payer												
	Formal	0.09	0.164	0.002–3.48	0.26	0.22	0.023–2.65	0.30	0.41	0.01–7.78	0.74	0.685	0.14–3.88
	Informal	0.13	0.117	0.009–1.92	0.16	0.007*	0.05–0.51	0.24	0.42	0.005–11.71	1.21	0.775	0.27–5.58
	Unemployed(ref)	1			1			1			1		
10	Income of the payer⊥												
	≤ $1,389 (N500,000)	289.3	<0.001*	29.03–3064.2	313.3	<0.001*	44.24–2218.6	277.22	<0.001*	49.89–1540.28	621.19	0.001*	35.00–11023.3
	>$1,389 (N500,000) (ref)	1			1			1			1		
11	Type of hospital												
	Private(ref)	1			1			1			1		
	Public	0.94	0.904	0.29–3.06	1.38	0.53	0.45–4.24	0.82	0.761	0.19–3.53	0.51	0.013*	0.312–0.833
12	Grade of Hospital												
	Secondary(ref)	1			1			1			1		
	Tertiary	3.69	0.033	0.0005–25.18	3.19	0.05*	1.00–10.16	2.92	0.226	0.44–19.20	3.74	0.113	0.68–20.65
13	Health Insurance												
	Yes	0.11	0.378	0.0005–25.2	0.17	0.001*	0.003–0.12	0.05	0.032*	0.003–0.71	0.12	0.080	0.011–1.37
	No (ref)	1			1			1			1		

AOR = Adjusted Odds Ratio; CI = 95%Confidence Intervals

*Significant Associations

⊥-Income UOR have wide CI due to the skewed distribution (Fig 3)

‡CS = Caesarian Section; # = Igbo, Hausa

At 5% threshold in the unadjusted analysis (Table 3), six variables were found to be significant. Non-slum dwellers were less likely to experience catastrophic expenditure compared to slum dwellers (UOR = 0.32, p = 0.021; 95%CI: 0.13–0.79). Younger payers aged 39 years and below were more likely to experience CHE compared to payers in the age group of above 40 years (UOR = 2.24, p = 0.018; 95%CI: 1.20–4.21) (Table 3). Payers that were currently married were more likely to experience CHE compared to those that were not currently married (UOR = 2.54, p = 0.024; 95%CI: 1.17–5.52) (Table 3). Payers with formal (UOR = 0.11, p = 0.013; 95%CI: 0.02–0.55) and informal jobs (UOR = 0.12, p = 0.064; 95%CI: 0.01–1.17) were less likely to experience CHE compared to unemployed payers. Payers with health insurance coverage were about 9 times less likely to experience CHE compared to payers without any form of health insurance coverage (UOR = 0.11, p = 0.023; 95%CI: 0.02–0.68). Payer’s income when less than N500,000 were more likely to experience CHE compared to income over N500,000 (UOR = 174.96, p < 0.001; 95%CI: 28.97–1056.94). This result was found to be statistically significant.

In the adjusted analysis (Table 4), only age of payer (AOR = 3.51, p < 0.005; 95%CI: 1.63–7.54) and income (AOR = 289.3, p < 0.001; 95%CI: 29.03–3064.2) were the signifcant predictors at 5% threshold.

Sensitivity analyses were carried out at cut-offs for CHE of 10%, 25% and 40%. At 10%, the proportion of slum dwellers and non-slum dwellers who experienced CHE was 71.7% and 36.3% respectively. At 25% the proportion of slum dwellers who experienced CHE were 66.5% and 21.6% for non-slum dwellers and at the 40% cut-off, 57.9% of slum dwellers experienced CHE compared to 15.4% for non-slum dwellers.

## Discussion

The study sought to compare the prevalence and characteristics of people that experienced CHE among urban slum dwellers and non-slum dwellers within a Nigerian metropolis. Findings from the study demonstrate that the burden of OOP payment for emergency surgery was substantial among both slum and non-slum dwellers, thus reflecting limited financial protection available for both groups. Notably though, CHE was found to be significantly higher among slum dwellers compared to non-slum dwellers. It has been reported in literature [35, 53] that access to social networks and social solidarity schemes which are often used to mitigate CHE are less available in the slums and have been documented as reasons for the level of CHE in the slum population. In addition to the lack of social solidarity schemes [54] to support households during emergency care, slums offer poor or no employment opportunities, amenities or earning capacity (income) [22, 55, 56]. The slum dwellers are not only disadvantaged in terms of an inadequate physical environment, but may not be able to save for health-related needs, thus rendering them ill-equipped to handle sudden health emergencies. In a recent study, this ad hoc savings culture was an occurrence reported by both slum and non-slum dwellers who experienced emergency surgery, however, as that was a qualitative study, the prevalence of not saving is not known [35].

The prevalence of CHE among slum dwellers (74.1%) and non-slum dwellers (47.7%) was quite high compared to other studies reported in the literature [8, 10, 15, 57]. Amakon and Ezenekwe (2012) found a 24% prevalence of CHE among the richest income quintiles in Nigeria while another study conducted in Kenya estimated the prevalence of CHE amongst its non-slum dwellers to be 23% [8, 10]. The higher prevalence observed in this study could be due to the fact that respondents in this study were patients scheduled for emergency surgery (which is not only a sudden occurrence but also financially demanding), differing considerably in terms of nature of care sought by the respondents in the Kenyan study that explored care related to seizures, difficult breathing, measles and injury. Considering also that this was a hospital-based study (in which the population recruited for this study are households that were scheduled for surgery) instead of community-based households and by virtue of their status being hospital-based respondents, the vulnerability to CHE is much higher already and as such the plausible explanation for why proportion of CHE was this high in our study. Extrapolation of study findings in comparison with other studies needs to be done bearing this difference in mind. The increased risk and vulnerability to CHE with hospitalization as seen in this study has important policy implications and has been also brought to the fore in a prior study [58]. An urgent need for innovative social or welfare packages for households faced with emergencies is urgently needed.

The significant statistical association between the setting of the respondents (whether slum or non-slum) and occurrence of CHE in this study is a contributory factor to earning disparities between the urban slum dwellers and their non-slum counterparts. The increased likelihood of slum dwellers to suffer CHE can be attributed to their relatively limited earning capacity. Slum-dwellers, in general, have lower income earning capacities [59], which in turn increases their susceptibility to CHE during illness episodes. The reduced income of slum dwellers also limits considerably the ability to raise funds through alternative sources as they cannot prove that they can pay back [60, 61]. Thus, the reduced earning capacity of slum dwellers increases the chances and risks of suffering CHE. The correlation between income and vulnerability to CHE is validated in this study. Health insurance is yet another factor that is very significant in reducing CHE in literature [62] and which is also observed in this study. The higher coverage of insurance seen among the slum dwellers compared to non-slum dwellers was an intriguing yet unsurprising finding. Popular local savings clubs [35] or community based health insurance schemes (CBHIS) [63, 64] that are commoner among slum regions might be accountable for this increase. In recent studies, a scale up of community based health insurance schemes has been stepped up to cater for health needs of informal populations–an innovation that has witnessed differing levels of acceptance and coverage in different settings [65, 66]. The functionality of these schemes is still in doubt with respect to the effectiveness of coverage, as we observe that CHE is still much lower among non-slum dwellers compared to the slum dwellers. The protective effect of health insurance from incurring CHE as observed in our study findings validates what is obtainable in literature [38, 67].

The indications for emergency surgery establish that pregnancy is a high-risk period when patients are at a much higher risk of experiencing CHE especially if they have to undergo a caesarian section that was initially unplanned. The continuous rise in the trend of CS at delivery, observed in the past 30 years, has been attributed to many factors such as improved screening abilities, bad medical histories [68] and physician-driven incentives to perform more CS, which is a perverse outcome of the pay for performance initiative [69, 70]. The mode of delivery is however, not the sole factor or period when CHE likelihood is higher but also the type of residence (slum or not, urban or rural). In a study conducted in Enugu Nigeria, a significantly lower odds of having CS was observed among women living in rural settings compared to residents living in urban settings [71]. These observed differences have been attributed to socio-cultural issues that influence variation and ultimately the acceptance of the procedure across Africa [72]. Despite these socio-cultural issues, CS has been pronounced key to reducing maternal mortality in Africa regardless, coupled with a massive scale-up of health systems [73]. Musgrove and colleagues (2000) further argue that the overall utilization and access to emergency obstetric care are not only dependent on perceived patient need but also the responsiveness of the system [74].

The involvement of spouses as payers for many of the CS surgeries is not only borne from the fact that Nigeria is patriarchal but equally emphasizes the role of men and their involvement in supporting the reproductive health of women that has been reported in the literature [75]. Our study illustrates that men take responsibility for the reproductive health of their spouses which corroborates the literature that has also been shown in mother to child transmission of HIV research [76]. Correlation between a higher incidence of CHE with female gender, private facility utilization in this study is supported by findings from Okedo-Alex et al. (2019) [77]. Although Cleopatra and Eunice (2018) found a reduced incidence of CHE with utilization of private facilities [58], a key driver why clients are more likely to experience CHE in private facilities is the relative higher cost at which healthcare services are provided in the quest to provide quality care [78].

As supported by the literature, the finding of an increased odds of catastrophic expenditure in those aged less than 40 years amongst both payers and patients can be explained by this being the childbearing years [79]. The increased odds of impoverishment associated with the unemployment rate is not surprising–as observed in higher thresholds of CHE. Finding that unemployment is linked to CHE in our study confirms what has been documented in the literature [80, 81]. Being the patient and also the payer greatly reduces the ability to seek assistance from social networks to assist with hospital bills in form of loans or monetary gifts. This may well explain the increased susceptibility to CHE that was established in this study.

The findings of this study must be interpreted bearing the following limitations in mind. Firstly, the effect of household sizes was not considered [51]. The approach that was used to estimate CHE, was that of proportionality of income [8] instead of estimating based on household expenditure. The proportionality of income was preferred in this study based on result findings from the pre-test; that showed a poor recall of household expenditure by respondents. Thus, findings from this study should be applied cautiously to studies in which CHE was calculated based on household expenditure that is equally an acceptable method of estimation [82–85].

However, our data collection period was at the point of admission that is before the payer had experienced CHE and so it is not likely that the degree of CHE was influenced by recall bias. This study was conducted in the southwestern part of the country alone where the dominant ethnicity is Yoruba and these findings may not apply to all of Nigeria. Invariably, comparison of study findings with other parts of the country such as the Northern and Southeastern parts of the country should be done cautiously, where the dynamics of socio-cultural beliefs and practices play out and interact differently from the southwest [86, 87]. The cross-sectional design of the study means that we cannot establish causality in this study [88]. The possibility of an underestimation of CHE cannot be ruled out because we excluded some payers that were also patients. The prevalence of CHE among payers who are also patients might differ from those where the payer is not the patient. We also excluded household payers that were not exclusively responsible for payment of the hospital bills. The study also could not ascertain what proportion of the participants were pushed into deeper poverty nor were we able to provide immiserization figures because this was outside the scope of the study. It would be desirable to explore this in further studies.

Nevertheless, our study brought to light important findings such as providing baseline information on the prevalence of catastrophic expenses among both slums and non-slum dwellers in a systematic analytic manner and illustrating a greater burden of CHE among the slum-dwellers. This study also provided extensive detail of the prevalence of CHE at four thresholds that have been suggested in literature. Furthermore, the study demonstrated that there were more significant factors at 25% threshold of CHE compared to other threshold levels and offers baseline data on further research among slum dwellers facing emergencies.

## Conclusion

Evidence from this study establishes a higher prevalence of catastrophic health expenditure among slum dwellers compared to non-slum dwellers when they undergo emergency care admissions that require surgery in health facilities. Reducing the effect of catastrophic health expenditure requires a feasible policy reform that focusses resources on mechanisms that increase financial protection for health care consumers of emergency services regardless of whether they are slum or non-slum dwellers. This reform is also required to address the unaffordability of emergency care that is common in the secondary and tertiary facilities of low- and middle-income countries.

A review of the current National Health Insurance Scheme (NHIS) to bridge gaps and strengthen the existing health insurance structure is also necessary to protect families and households from exorbitant and catastrophic health care costs–a common consequence of seeking emergency surgical attention in slum and non-slum areas. Increased funding to the health sector and poverty alleviation schemes, in slums, in particular, are equally desirable to achieve a positive impact on the burden of paying for emergency surgeries and on the general health of the populace going forward.

## List of abbreviations

CARTA: Consortium for Advanced Research Training in Africa
CHE: Catastrophic Health Expenditure
FSSHIP: Formal Sector Social Health Insurance
LGA: Local Government Areas
LOS: Length of Stay
OOP: Out of Pocket
OOPE: Out of Pocket Expenditure
NHIS: National Health Insurance Scheme
WHO: World Health Organization
